# Case report of congenital myotonic dystrophy with multiple prenatal sonographic findings

**DOI:** 10.1515/crpm-2023-0029

**Published:** 2024-04-29

**Authors:** Zita Strelcovienė, Eglė Machtejevienė, Meilė Minkauskienė, Rasa Traberg

**Affiliations:** Medical Academy, Department of Obstetrics and Gynaecology, 230647 Lithuanian University of Health Sciences, Kaunas, Lithuania; Medical Academy, Department of Genetics and Molecular Medicine, 230647 Lithuanian University of Health Sciences, Kaunas, Lithuania

**Keywords:** arthrogryposis, congenital myotonic dystrophy, macrocephaly and ventriculomegaly, pathology of diaphragm, polyhydramnios

## Abstract

**Objectives:**

Myotonic dystrophy 1 (DM1) is an autosomal dominant inherited neuromuscular disorder. The most severe form is congenital myotonic dystrophy (cDM). Prenatal diagnosis is complicated and sonographic findings of cDM that are not pathognomonic occur in the late second or early third trimester of pregnancy.

**Case presentation:**

It is the case of prenatally diagnosed cDM. In 32 weeks of pregnancy multiple sonographic findings such as severe polyhydramnios, bilateral talipes, fetal legs akinesia, macrocephaly with mild bilateral ventriculomegaly, right-sided pleural effusion and diaphragmatic pathology were observed by fetal medicine specialist. As the patient complained of weakness in her limbs, she was consulted by a neurologist. The neurological examination revealed a pathognomonic sign of DM1 – grip myotonia. The amniotic fluid and the mother’s blood sample were further tested for DM1. This identified >150 repeats in one copy of the DMPK gene of the both, which is consistent with the diagnosis DM1.

**Conclusions:**

The sonographic findings of fetal limb abnormalities with progressive polyhydramnios is an indication for maternal neurological examination and genetic testing due to myotonic dystrophy.

## Introduction

Myotonic dystrophy type 1 is an autosomal dominant inherited neuromuscular disorder. The incidence of myotonic dystrophy 1 (DM1) is about 1 in 8,000 in Europe [1, 2]. However, 2021 study from the newborn screening program in the state of New York, have found CTG repeats expansion 50 are more often – 1 per 2,100 births [3].

Steinert first described this disease in 1909 and the gene responsible for DM1 was elucidated in 1992. DM1 occurs due to a mutation in the DMPK (myotonic dystrophy protein kinase) gene located on chromosome 19q13.3, when the unstable CTG trinucleotides repeat 50 times and more. The transcription of the expanded DNA results in the mutant RNA, that causes the splicing defects in some proteins: CUG binding protein and musclebind-like protein, which is abundant in skeletal and in brain tissues. Healthy person has five to 37 the CTG copies. The higher the number of repeats is detected, the earlier the disease manifests and the more severe the clinical picture of DM1 is. The anticipation phenomenon is typical for DM1, since this condition is more severe in successive generations [4, 5].

The clinical picture of DM1 depends on the severity of disease. In mild and classical forms patients usually complaint of the weakness in limbs, voice and pronunciation alterations, myotonia, and fatigue or sleepiness. Also, endocrinopathies (hyperinsulinism, hypothyroidism, and testicular atrophy), gastrointestinal motility impairment and cardiac disorders can develop [6]. Neurological examination is crucial in neuromuscular disease as grip myotonia (the delayed relaxation of finger after gripping) is a characteristic sign even in the early stages of DM1 [7]. Congenital DM is the most severe form. Due to generalized muscle hypotonia the newborn has respiratory difficulty, which can cause up to 30–40 % infants’ deaths in the neonatal period. The survivors have feeding problems, delayed motor development and mental retardation [8].

Prenatal diagnosis of cDM is challenging, especially when family history is unknown. In the majority of cDM cases ultrasounds findings such as severe polyhydramnios and fetal limb anomalies might occur late in pregnancy, and thus the specialist must be vigilant to suspect congenital neuromuscular disease of the fetus [7, 9, 10].

The aim of this article is to present the case report of congenital myotonic dystrophy with multiple sonographic findings during pregnancy, since no similar case of cDM was reported in the literature.

## Case presentation

**Figure 1:**
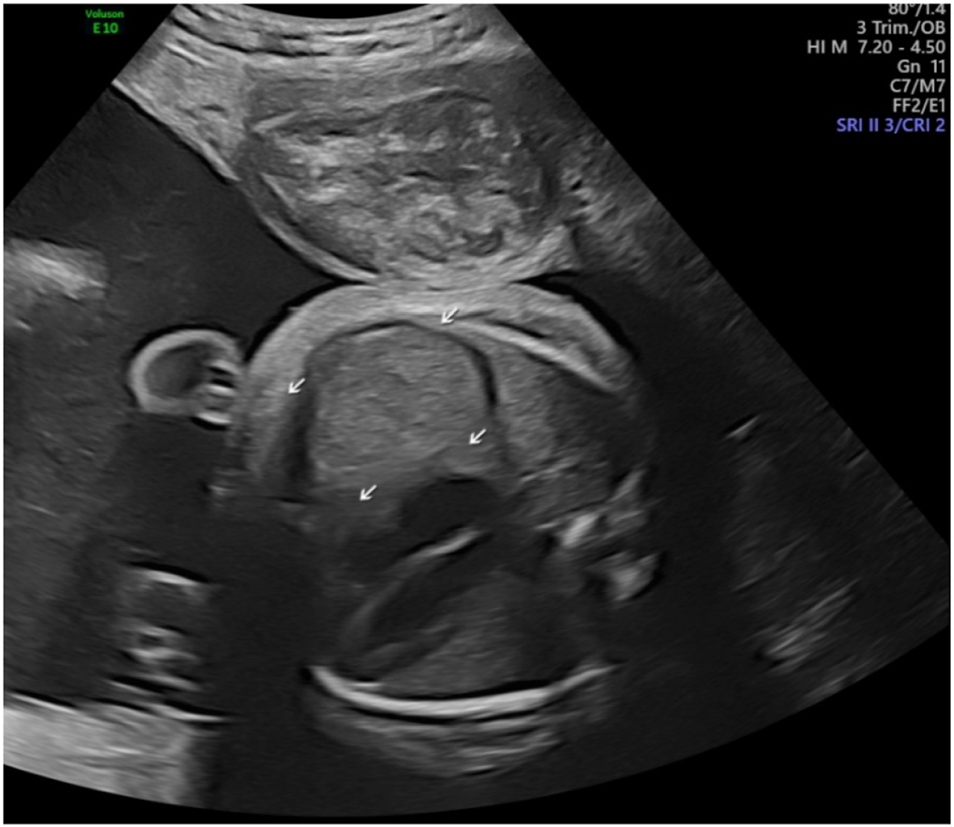
Prenatally the right-side diaphragmatic eventration was suspected and differentiated from diaphragmatic hernia at 30 weeks.

A 38-year-old G3P2 white woman was periodically consulted in a tertiary care center due to a high-risk pregnancy. She has one daughter and now has conceived after an *in vitro* fertilization procedure. There was an increased biochemical risk of the 21 trisomy (1:75), but a non-invasive prenatal testing (NIPT) was reported as low risk, male. The first and second trimester anomaly ultrasound scans were performed and did not show any signs of fetal malformation. At 30 weeks, she was admitted to the hospital because of a suspected intrahepatic cholestasis. During ultrasound scan, polyhydramnios was revealed and a right-side fetal diaphragmatic hernia was considered. Two weeks later, the woman was investigated by an experienced fetal medicine specialist. During this appointment, the patient complained of reduced fetal movements and also noticed, that the fetus was less active compared to the first one. Ultrasound examination showed severe polyhydramnios (the deepest vertical pocket was 142 mm), bilateral talipes, a fetal legs akinesia – arthrogryposis, a macrocephalia with mild bilateral ventriculomegaly, a right-sided pleural effusion and a diaphragmatic eventration or hernia (Figures 1 [Fig j_crpm-2023-0029_fig_002] [Fig j_crpm-2023-0029_fig_003] [Fig j_crpm-2023-0029_fig_004]–5). Due to severe symptomatic polyhydramnios, she was treated in the obstetric department and an amnioreduction was performed. The patient had complaints of hand weakness and fatigue in legs for many years. Because of neuromuscular disorder suspicion the patient was referred to the neurologist consultation. Neurological examination showed a pathognomonic sign of DM1-the grip myotonia. According to past medical records and maternal neurological examination (a grip myotonia, a mild myopathic face with sunken cheeks and drooping eyelids were found) and prenatal ultrasound findings, the myotonic dystrophy 1 type to both – woman and fetus – was suspected. The sample of the amniotic fluid was further tested for CDM1. This identified >150 repeats in one copy of the fetal *DMPK* gene, consistent with a diagnosis of CMD. The blood sample of the mother also confirmed expanded repeats >150 in her *DMPK* gene. Genetic laboratory was able only to detect as many as 150 CTG repeats in fetus and woman samples. The exact repeats of CTG are unknown due the laboratory facility.

**Figure 2: j_crpm-2023-0029_fig_002:**
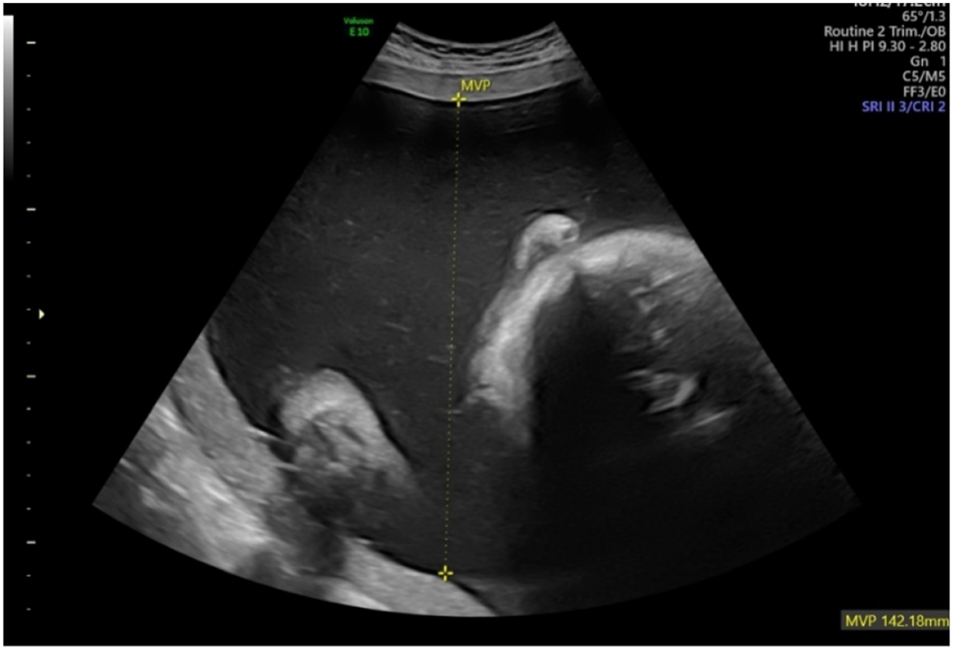
Polyhydramnios at 32 weeks. (deepest vertical pocket 142 mm).

**Figure 3: j_crpm-2023-0029_fig_003:**
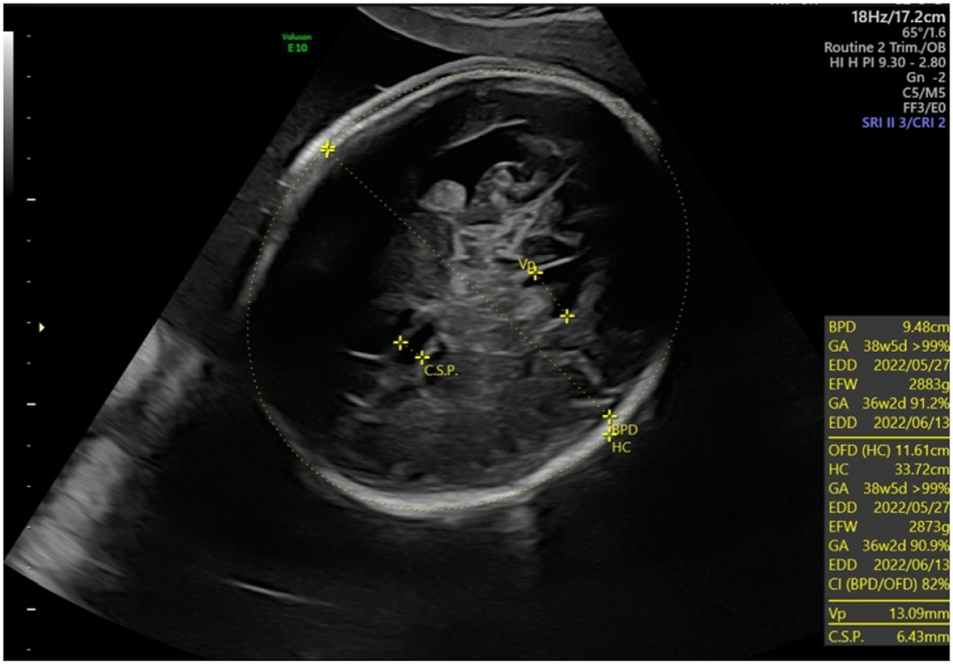
Macrocephaly (head circumference +3 SD) and ventriculomegaly (lateral ventricles 13 mm) for the fetus with cDM at 32 weeks.

**Figure 4: j_crpm-2023-0029_fig_004:**
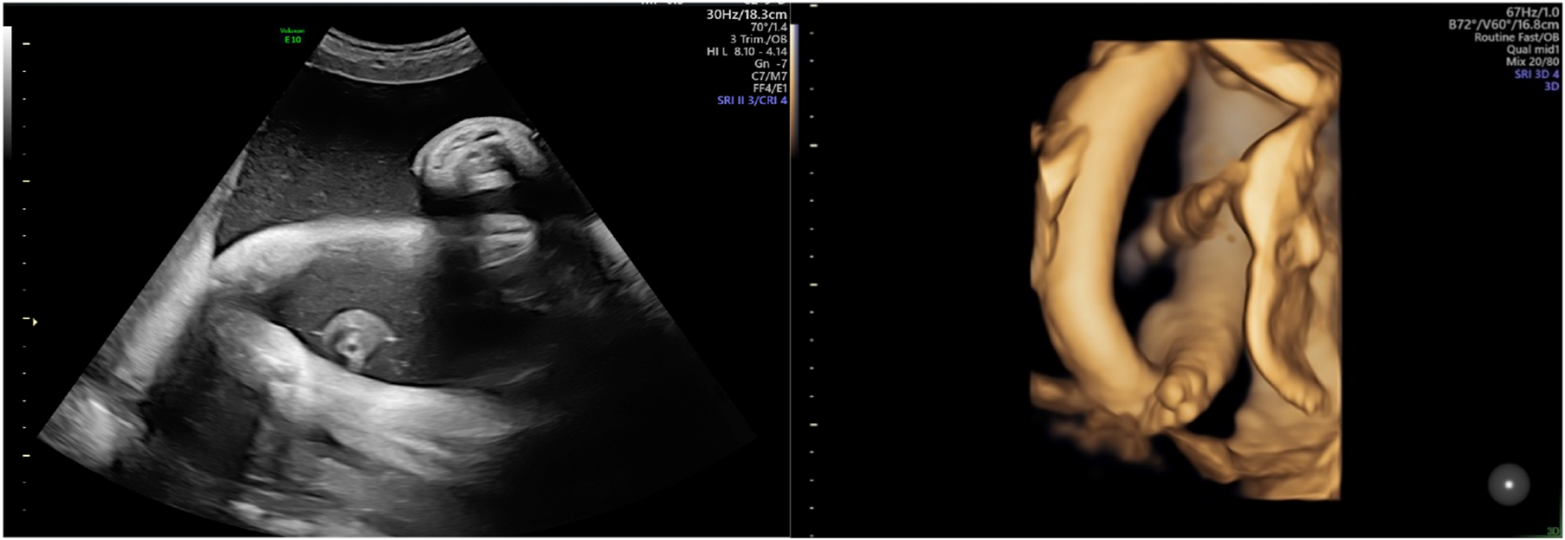
Decreased fetal movements with extended fetal lower limbs and rotated foot on 2D and 3D for the fetus with cDM at 35 weeks.

**Figure 5:**
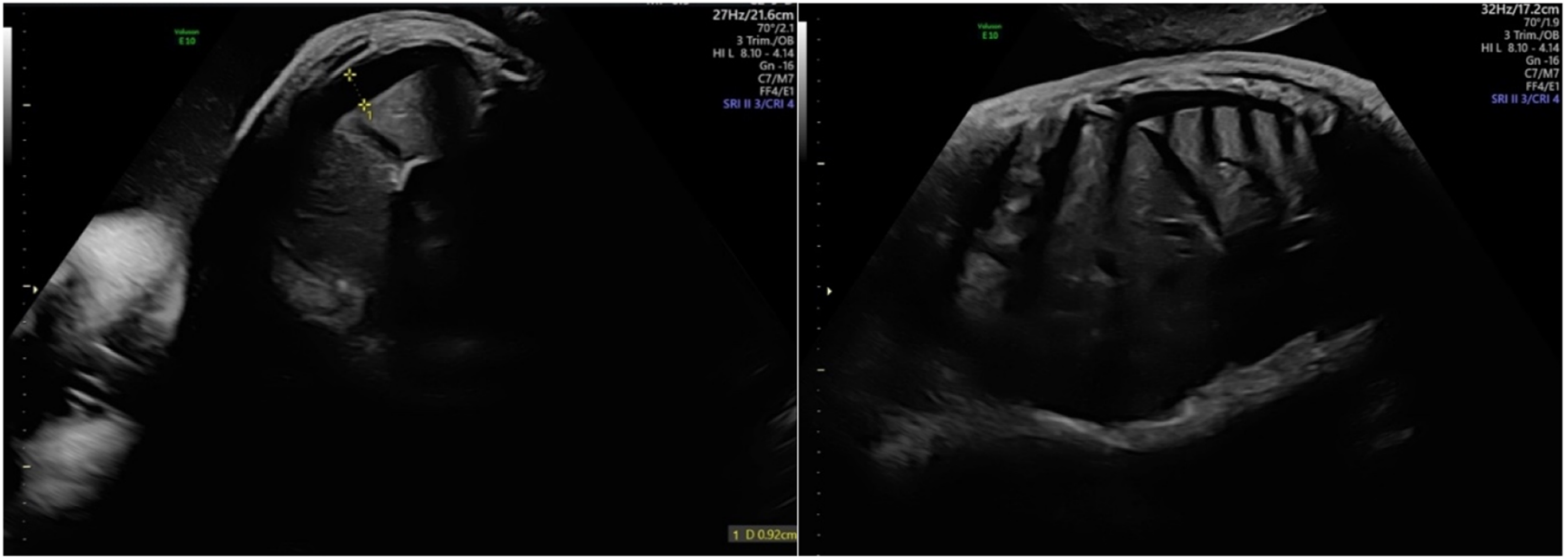
Right-sided pleural effusion on transverse and sagittal planes for the fetus with cDM at 35 weeks.

The baby was born in the 36th week of pregnancy by elective caesarean section because of a breech presentation. The newborn was a 3,590 g male with no respiratory efforts, had general cyanosis and no tone, bradycardia of 60 bpm with APGARs of one at 1st, 5th and 10th minutes. Despite he was intubated in the 5th minute of life, normal breathing could only be ensured after 10 min of his life. At birth, the physical examination revealed bilateral talipes, extreme hip flexion and knee extension and tented mouth. The head circumference was 40 cm (100th percentile). All these features are clinically consistent with the prenatal diagnosis of cDM. After admission to the neonatal intensive care unit, palliative ventilation and nutritional treatment was provided. A tracheostomy and gastrostomy were placed. Other symptoms such as congenital hydrocephalus and bilateral hydronephrosis do not require treatment. The chest and abdominal radiographs did not confirm any congenital pathology of the diaphragm. In addition, the right-sided pleural effusion disappeared spontaneously. The infant spent 56 days in NICU and then was transported to Pediatric intensive care unit for palliative care. Due to the main disease, the motor and mental development of the child is severely impaired, requiring intensive rehabilitation measures. At age of six month of the patient bilateral sensorineural deafness was diagnosed. After the hearing aids were fitted, the boy began to react more actively to his environment.

As DM1 is autosomal dominant disorder, clinical geneticist recommended to evaluate the patient’s first-degree relatives. The patient’s father had slight ptosis and slight frontal balding. Genetic testing of DMPK gene showed 70 CTG repeats that confirmed mild DM1. The patient’s brother presented with grip myotonia, muscle weakness. His *DMPK* gene testing showed >150 CTG repeats and confirmed classical DM1. Patient’s 13 years old daughter presented with slight learning disability, muscle weakness and grip myotonia. Classical MD1 was confirmed after DMPK gene testing.

## Discussion

Congenital DM can be easily diagnosed prenatally if the couple is aware of familial DM1. Targeted tests (chorionic villus sampling, amniocentesis or cordocentesis) can be performed, and preimplantation genetic testing is also indicated for the family [11]. Otherwise, the sonographic findings of cDM are not pathognomonic and only appear late in the pregnancy. In our case, cDM was suspected at 32 weeks with multiple sonographic findings.

The first sonographic sign in present case was an observed congenital pathology of the diaphragm accompanied by polyhydramnios. Recently, Pezolli and colleagues presented a case report of congenital diaphragmatic hernia with the primary condition of cDM, diagnosed only postnatally [12]. In our case, the diaphragmatic pathology on the right side was suspected prenatally but not confirmed postnatally. It could therefore be a transient condition related to changes in the skeletal musculature of the diaphragm.

Polyhydramnios is one of the most frequently identified pathologies associated with cDM [7, 9, 10]. In majority of genetic condition polyhydramnios occurs as a result of reduced or absent fetal swallowing. Polyhydramnios is usually severe and requires an amnioreduction as the mother suffers from respiratory distress, and medical seeking to avoid premature delivery [13], [14], [15].

Only at 32 weeks of gestation fetal lower limbs abnormality were detected during comprehensive ultrasound examination. According to the literature, in general the prenatal detection rate of arthrogryposis and talipes is insufficient [16]. However, arthrogryposis also is not a specific finding of cDM. More than 400 diseases are associated with abnormal limb positioning, and in 30 percent of cases it is caused by genetic conditions [17, 18].

Recent studies suggest that macrocephaly with or without borderline to mild ventriculomegaly in the third trimester may be a new sonographic finding associated with cDM, especially when head circumference was measured as normal on previous ultrasound examinations [19]. Regev et al. were the first to establish the association between neonatal mild ventriculomegaly and cDM [20]. Garcia-Alix A et al. reported the association of cDM with macrocephaly in ten of 14 newborns in 1991 [21]. Shinar et al. reported four cases of prenatal macrocephaly, two of which had mild ventriculomegaly. Prenatal macrocephaly is not secondary due to cerebral ventriculomegaly but may be due to prenatal megalocephaly caused an alteration in the DMPK gene [19].

The last sonographic finding was a right-sided pleural effusion, which disappeared spontaneously after birth. A retrospective cohort study showed that chylothorax could be one of the cDM symptoms, as the prevalence rate of chylothorax was higher in group of infants with cDM than without this condition [22].

## Conclusions

Prenatal diagnosis of congenital myotonic dystrophy is challenging, especially in the presence of a negative genetic history. The sonographic finding of fetal limb anomalies in combination with progressive polyhydramnios in pregnancy should indicate an investigation of congenital myotonic dystrophy. The patient should also be tested for grip myotonia. Other prenatal findings such as pleural effusion, pathology of diaphragm, macrocephaly with or without borderline ventriculomegaly may observed in case of cDM.
